# Supplementary material for: Mechanical Unfolding of Proteins—A Comparative Nonequilibrium Molecular Dynamics Study
Source: Biophys J. 2020 Aug 6;119(5):939–49. doi: 10.1016/j.bpj.2020.07.030 (PMC7474207; doi:10.1016/j.bpj.2020.07.030)
Supplement: Document S1. Figs. S1–S6 [file mmc1.pdf]

Biophysical Journal, Volume *119*

Supplemental Information

Mechanical Unfolding of Proteins—A Comparative Nonequilibrium Molecular Dynamics Study

Vasyl V. Mykuliak, Mateusz Sikora, Jonathan J. Booth, Marek Cieplak, Dmitrii V. Shalashilin, and Vesa P. Hytönen

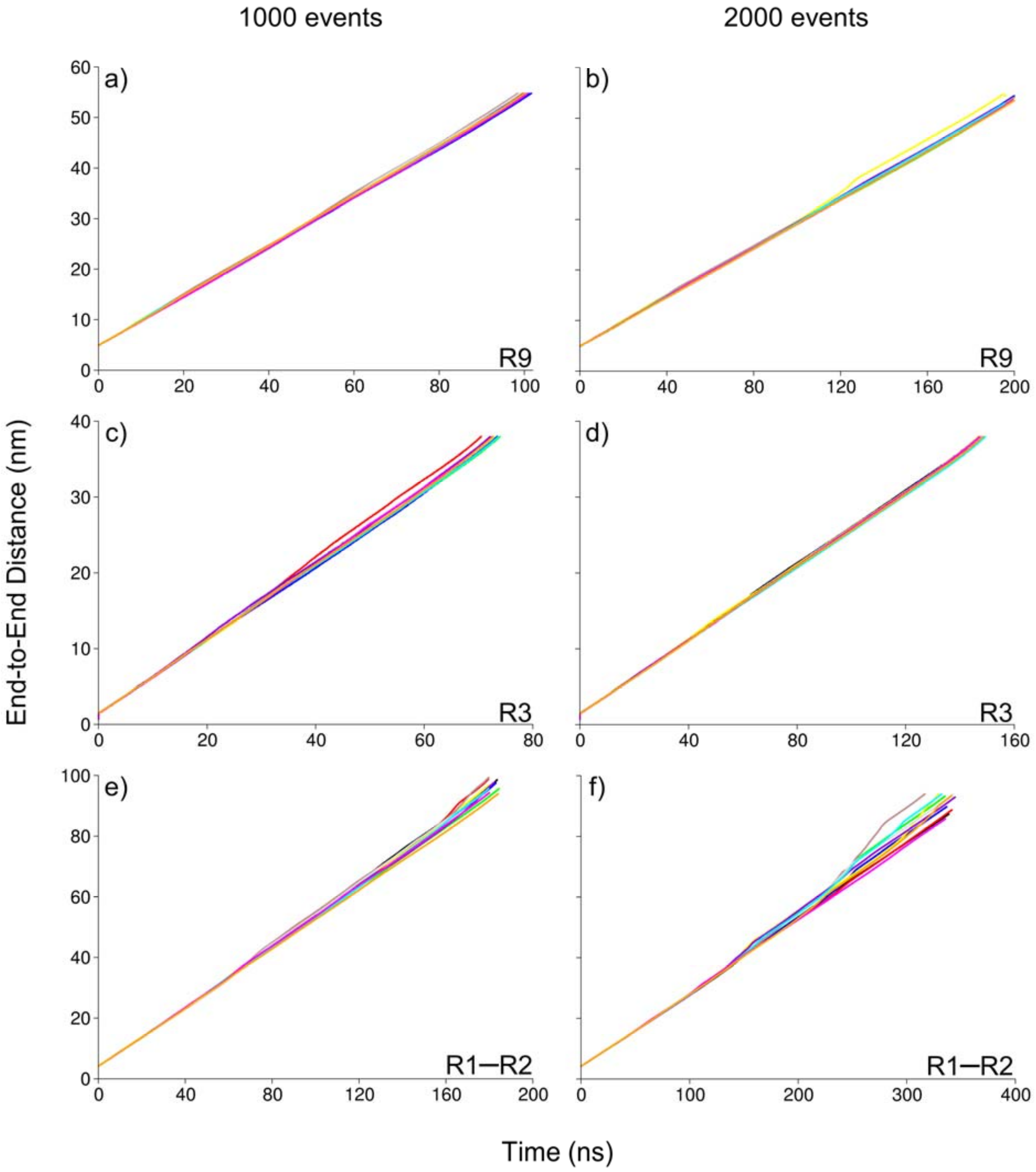

Figure S1. End-to-end distance as a function of time for the talin rod monomers (a & b) R9 and (c & d) R3, and (e & f) the tandem fragment R1–R2 during unfolding in BXD simulations, using (a, c & e) 1000 events and (b, d & f) 2000 events.

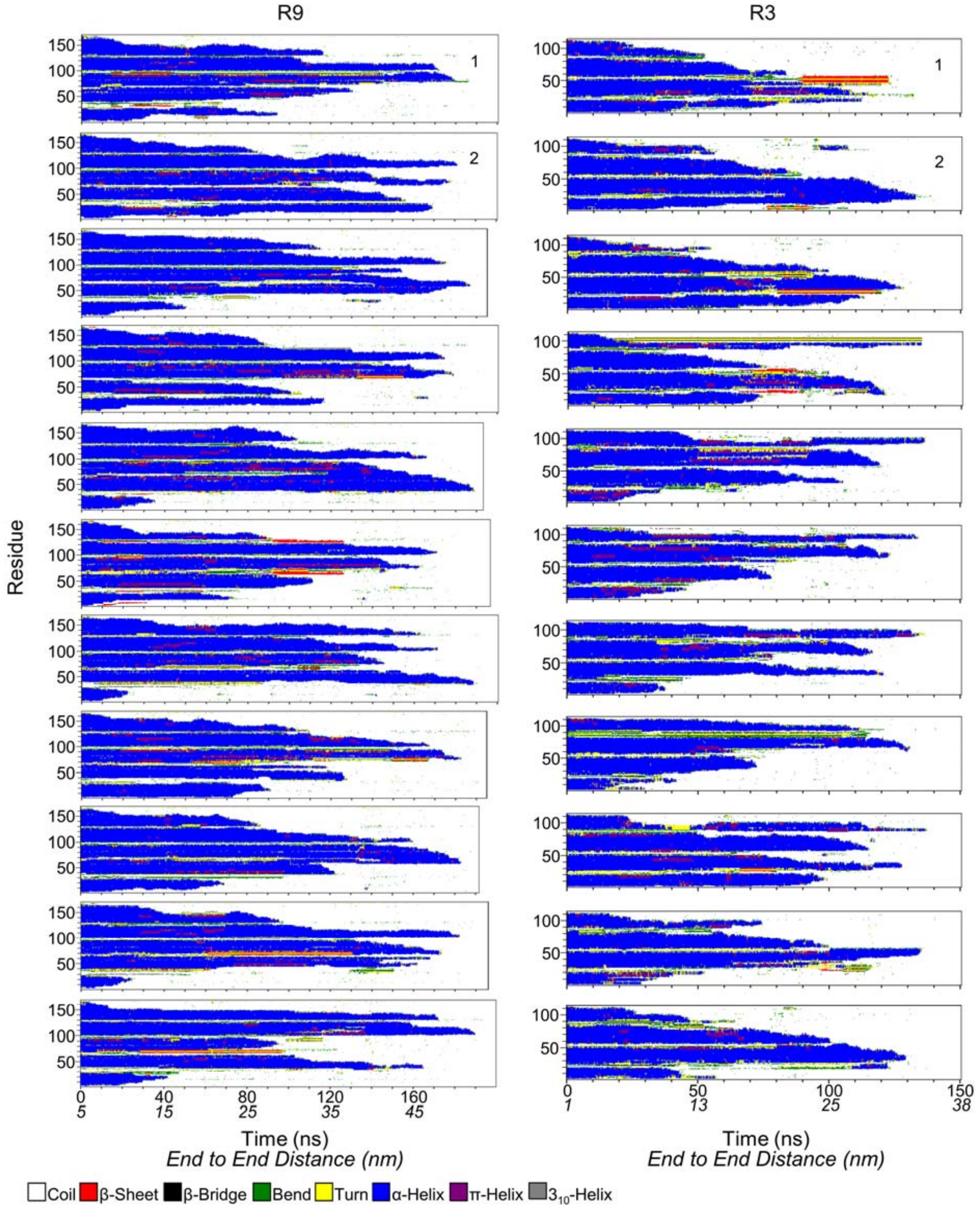

Figure S2. Secondary structure analysis of talin rod (left) R9 and (right) R3 during unfolding in BXD simulations. The analysis was performed using DSSP, for all eleven replicas.

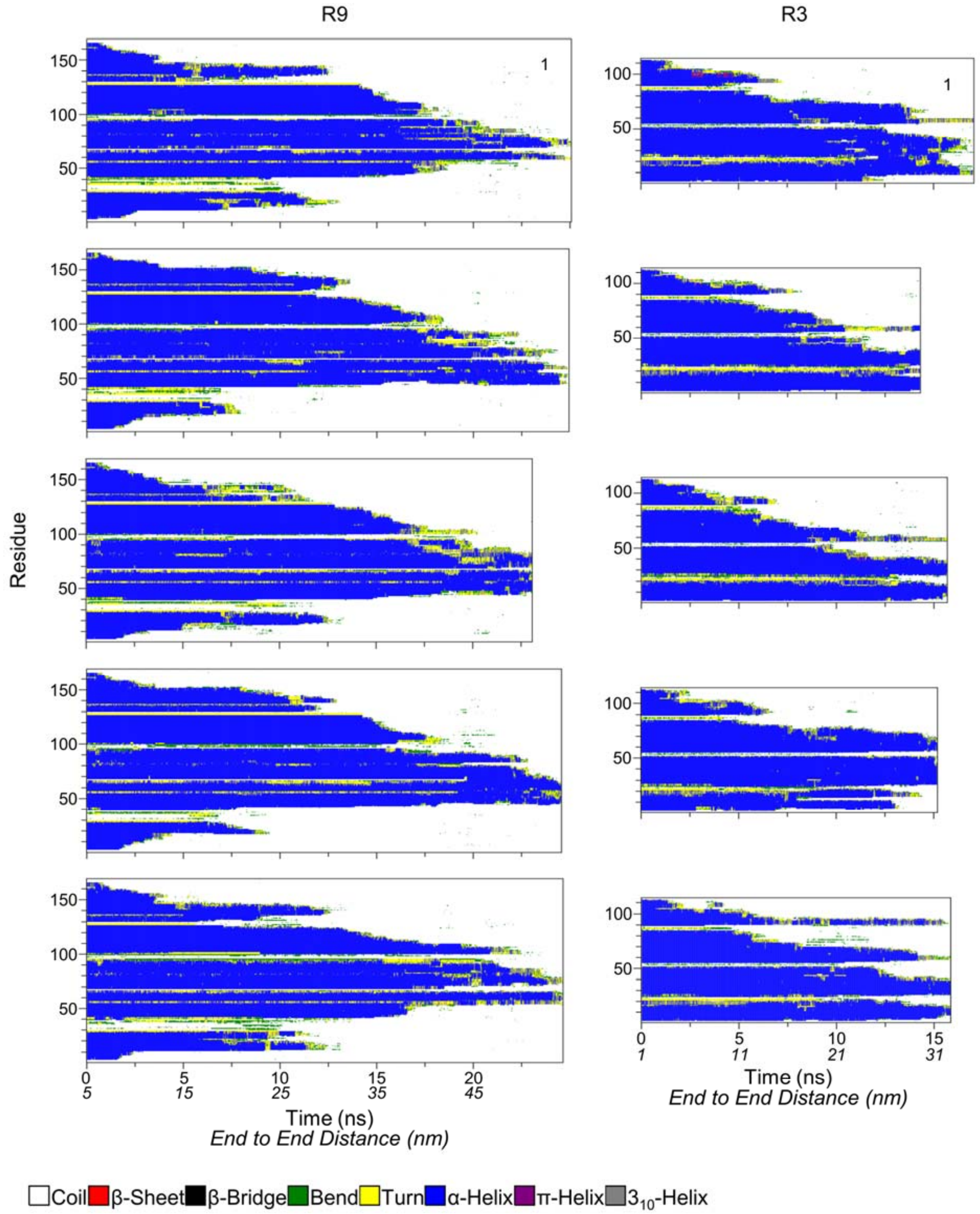

Figure S3. Secondary structure analysis of talin rod (left) R9 and (right) R3 during unfolding in all-atom SMD simulations. The analysis was performed using DSSP, for all five replicas.

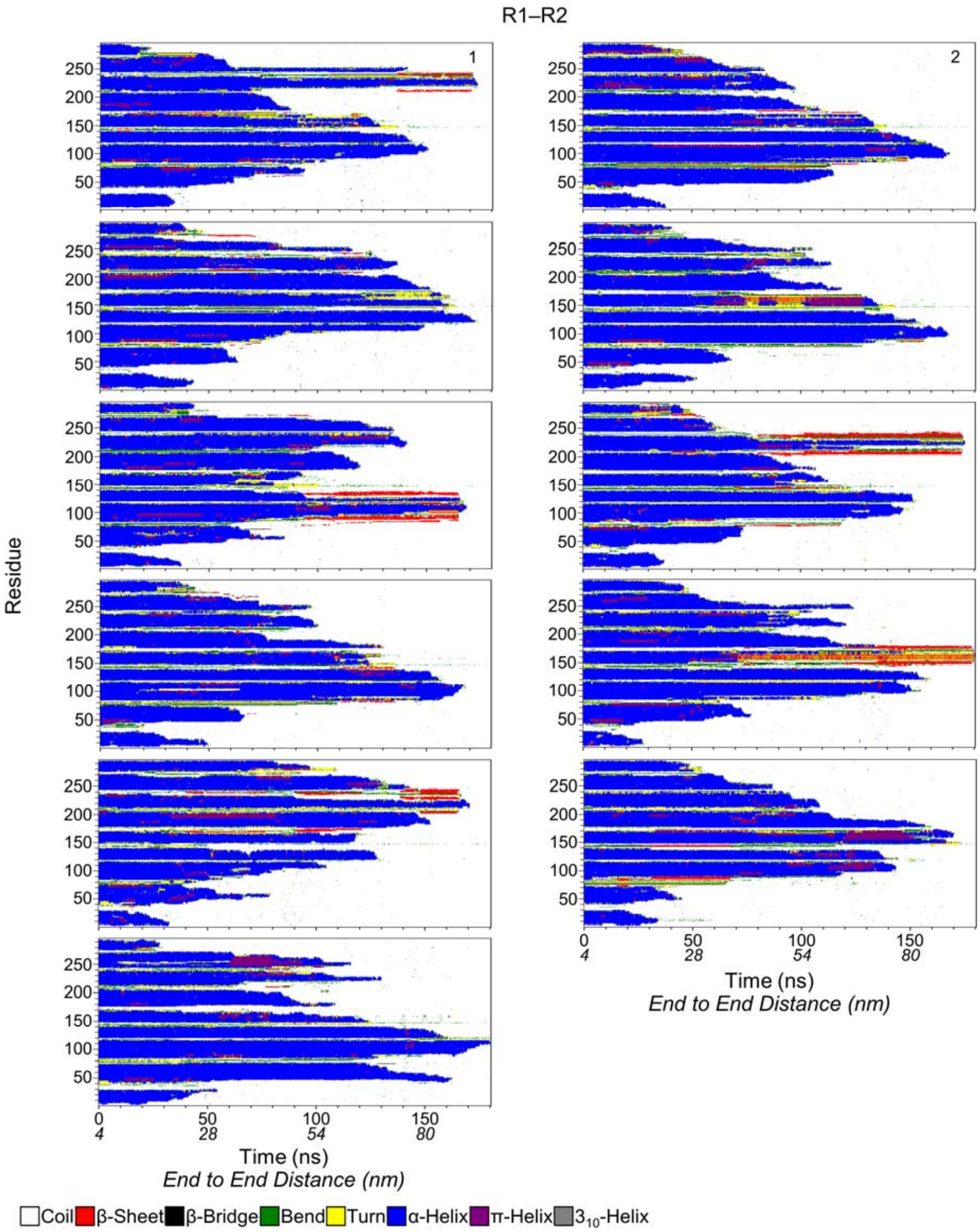

Figure S4. Secondary structure analysis of talin rod R1–R2 fragment during unfolding in BXD simulations. The analysis was performed using DSSP, for all eleven replicas.

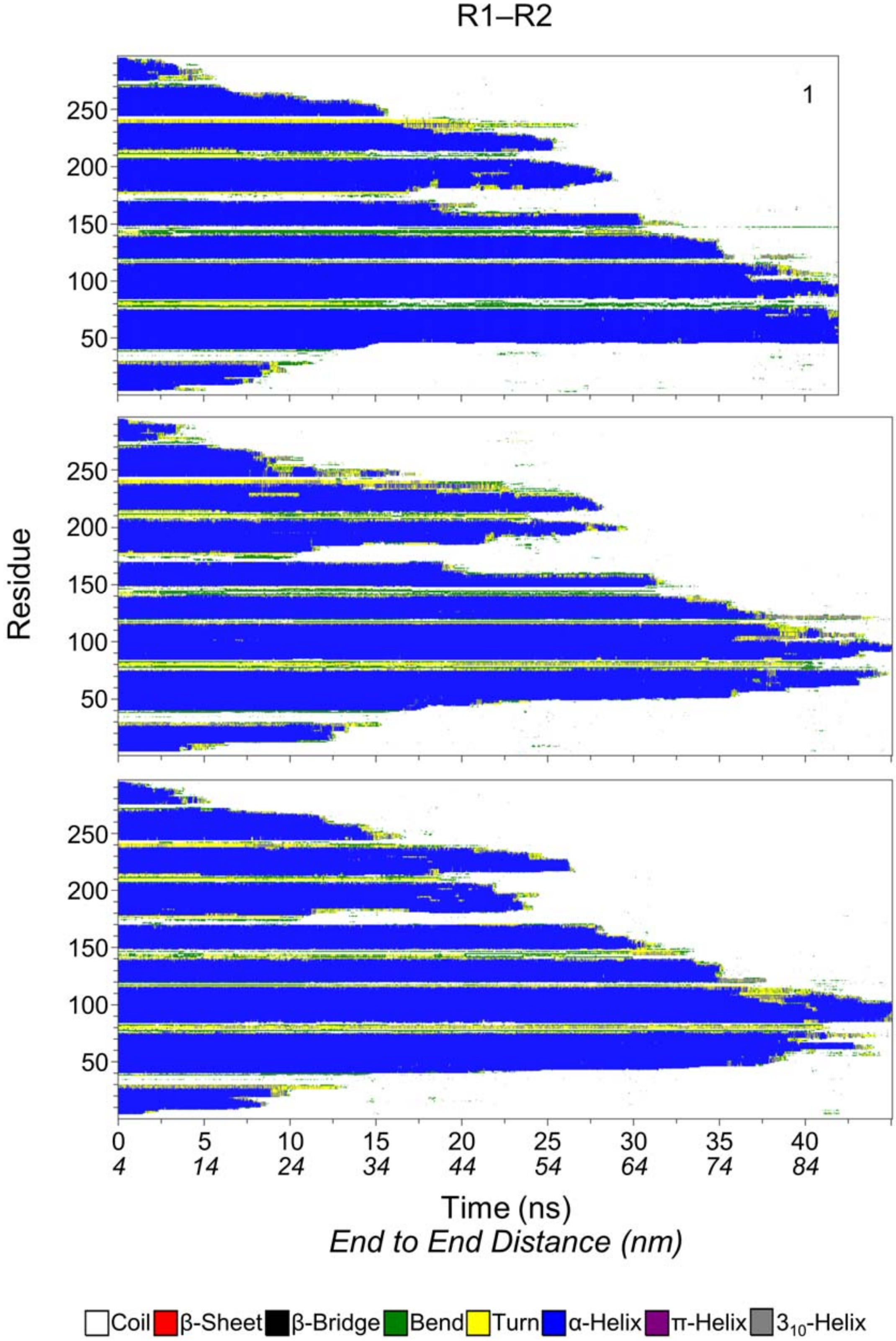

Figure S5. Secondary structure analysis of talin rod R1–R2 fragment during unfolding in all-atom SMD simulations. The analysis was performed using DSSP, for all three replicas.

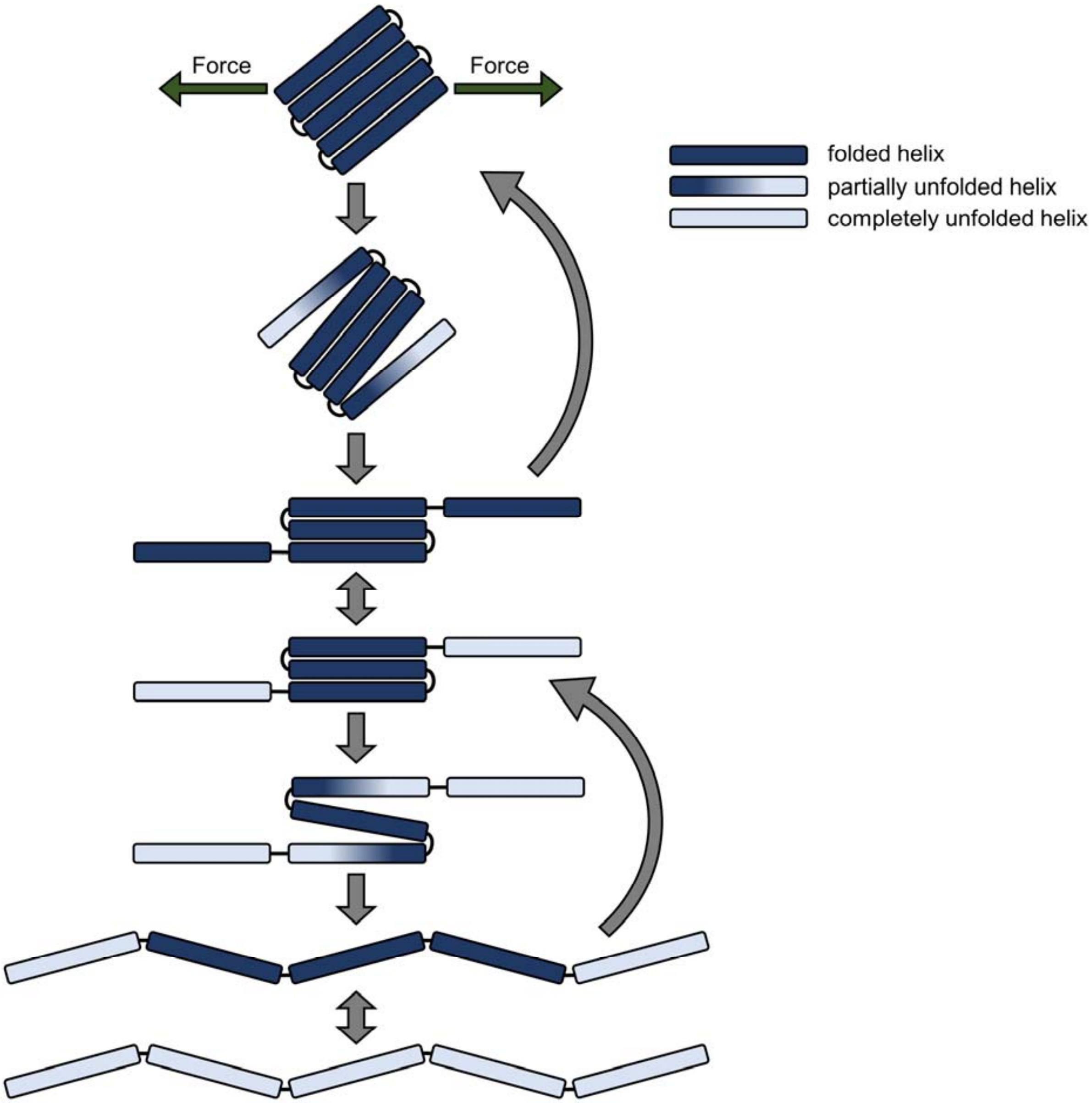

Figure S6. A model unfolding of the individual 5-helix talin rod subdomain. In the absence of force, the subdomain is completely folded. Under force, the terminal helices (namely, H1 and H5) undergo partial unfolding during breaking of the 5-helix structure, but after dissociation from the remaining 3-helix state the secondary structure refolds. As the force increase, the terminal helices undergo complete unfolding, followed by the break of the 3-helix state in the same scenario. In case of force decreasing, the structure refolds.
