# Supplementary material for: Mechanical Unfolding of Proteins—A Comparative Nonequilibrium Molecular Dynamics Study
Source: Biophys J. 2020 Aug 6;119(5):939–49. doi: 10.1016/j.bpj.2020.07.030 (PMC7474207; doi:10.1016/j.bpj.2020.07.030)
Supplement: Document S2. Article plus Supporting Material [file mmc2.pdf]

Biophysical Journal
Article

Biophysical Society

Mechanical Unfolding of Proteins—A Comparative Nonequilibrium Molecular Dynamics Study

Vasyl V. Mykuliak,[1,2] Mateusz Sikora,[3] Jonathan J. Booth,[4] Marek Cieplak,[5] Dmitrii V. Shalashilin,[4] and Vesa P. Hytönen[1,2,*]
[1]Faculty of Medicine and Health Technology and BioMediTech, Tampere University, Tampere, Finland; [2]Fimlab Laboratories, Tampere, Finland; [3]Max Planck Institute of Biophysics, Frankfurt am Main, Germany; [4]School of Chemistry, University of Leeds, Leeds, United Kingdom; and [5]Institute of Physics, Polish Academy of Sciences, Warsaw, Poland

ABSTRACT Mechanical signals regulate functions of mechanosensitive proteins by inducing structural changes that are determinant for force-dependent interactions. Talin is a focal adhesion protein that is known to extend under mechanical load, and it has been shown to unfold via intermediate states. Here, we compared different nonequilibrium molecular dynamics (MD) simulations to study unfolding of the talin rod. We combined boxed MD (BXD), steered MD, and umbrella sampling (US) techniques and provide free energy profiles for unfolding of talin rod subdomains. We conducted BXD, steered MD, and US simulations at different detail levels and demonstrate how these different techniques can be used to study protein unfolding under tension. Unfolding free energy profiles determined by BXD suggest that the intermediate states in talin rod subdomains are stabilized by force during unfolding, and US confirmed these results.

SIGNIFICANCE Talin is an example of mechanosensitive proteins that undergo unfolding under force, which regulates their ability to bind different partners. We applied different nonequilibrium molecular dynamics (MD) simulation techniques to study unfolding of individual talin rod subdomains under tension. Specifically, we combined boxed MD, steered MD, and umbrella sampling and demonstrated how these methods can be combined when applied at different conditions from coarse-grained force field and implicit solvent to all-atom resolution with explicit solvent model. We provide a detailed analysis of talin rod subdomain unfolding, which suggests that the three-helix intermediate is stabilized by force during unfolding.

INTRODUCTION

Mechanical force has been shown to regulate functions of mechanosensitive proteins. Mechanical signals are converted to biochemical activity by modulation of protein structural states, which leads to a change in affinity for different binding partners; thus, functions vary depending on the structural state. Mechanical force applied to protein structure helps to overcome large free energy barriers between different structural states with different functions (1,2).

One such mechanosensitive protein is talin. It is an intracellular protein that physically connects the intracellular actin cytoskeleton to the extracellular matrix via transmembrane integrin receptors (3,4). It consists of a head domain, a large α-helical rod, and a dimerization domain. The rod domain has multiple binding partners, which apply mechanical load to its structure (5). Talin consists of 13 subdomains (R1–R13), usually called bundles, which are formed by four or five α-helices, packed into a chain. The ability of the rod subdomains to bind different partners is regulated by mechanical force. Folded subdomains bind F-actin, RIAM, DLC1, KANK1, and others, whereas vinculin is known to bind to unfolded subdomains (6–8). Thus, mechanical load applied to the talin rod structure modulates its functions by changing the affinity of binding sites for protein partners. Mechanical stretching of the talin rod leads to the unfolding of its subdomains, the opening of cryptic vinculin-binding sites, and the recruitment of vinculin, which also binds actin filaments (9–11).

In our previous studies, we have shown that talin rod subdomains have varying mechanical stability. The five-helix R9 is mechanically the strongest (12), whereas the four-helix R3 is known to be mechanically the weakest bundle

Submitted January 27, 2020, and accepted for publication July 23, 2020.
*Correspondence: vesa.hytonen@tuni.fi
Editor: Alexander Dunn.
https://doi.org/10.1016/j.bpj.2020.07.030
© 2020 Biophysical Society.
This is an open access article under the CC BY license (http://creativecommons.org/licenses/by/4.0/).

because of a cluster of four threonine residues in the hydrophobic core of the bundle (13,14). We demonstrated that under mechanical load, both five- and four-helix subdomains unfold through three-helix intermediates and hypothesized that the three-helix intermediates exist under force only (15). However, the free energy landscapes for the unfolding of the subdomains and thus free energy barriers and stability of the three-helix states remain unknown.

Unfolding of α-helical proteins, including the talin rod, has been previously studied using experimental and theoretical techniques. Experimental methods involve magnetic tweezers and atomic force microscopy (AFM), whereas theoretical methods utilize nonequilibrium molecular dynamics (MD) simulations techniques (16). One such technique is steered MD (SMD), which allows protein unfolding to be studied usually by extending the protein end-to-end distance using constant velocity or constant force pulling. Another technique is boxed MD (BXD), which divides the reaction coordinate into small boxes where the velocity of the trajectory is inverted in the direction of the reaction coordinate every time the trajectory hits a boundary of the box (17). The simulations can be performed at different detail levels using either explicit or implicit water model and all-atom, united-atom, or coarse-grained force field. These methods require careful parametrization, and the interpretation of the data is not straightforward. Moreover, there are a limited number of studies comparing the nonequilibrium methods in studying complex protein systems. It is worth noting that α-helical proteins are more challenging in unfolding experiments compared to β-structures because of the fact that α-helical proteins are softer.

In this study, we combined BXD and SMD simulations to study the mechanisms of unfolding for the individual talin rod subdomains R9 and R3 and tandem domains R1–R2. Combining the advantages of different nonequilibrium MD techniques, we propose a model of how these computational methods can be utilized to study proteins under mechanical load. We provide free energy landscapes of the unfolding using BXD and umbrella sampling (US), which demonstrates that the three-helix intermediates in talin rod subdomains exist under mechanical load only.

Theory

MD simulation helps to interpret experimental data on protein unfolding. SMD performs such simulations in the most straightforward way by pulling the protein termini (15,18). In SMD simulations, the pulling work is calculated, yielding a work profile along the reaction coordinate, which is similar to the potential of mean force (PMF) or free energy along the protein direction of pulling as a function of the displacement:

$$\Delta G \approx \Delta W \quad (1)$$

However, SMD simulation of the dynamics of an AFM unfolding experiment with realistic pulling speed would require too much computational time. In practice, the pulling speed in the simulations is several orders of magnitude faster than in an experiment. The pulling force used in SMD is also significantly higher than the experimental one. The use of nonphysical conditions, such as high force and high speed, may lead to the generation of artificial structural states.

BXD is another method that has recently been applied to simulations of protein pulling. BXD does not apply any additional pulling force. Instead, it splits the reaction coordinate into a number of boxes in which the trajectory is locked. When the trajectory "hits" a box wall, it is reversed with respect to the direction of the reaction coordinate. Once sufficient statistics are accumulated, the trajectory is allowed to the next box, and the procedure is repeated. The rate constant of going from box to box is calculated as an average time between the subsequent "hits." At the end, BXD produces a set of box-to-box rate constants from which PMF can be recovered. Then, the free energy difference between two neighboring boxes and the whole PMF profile can be recovered as

$$\Delta G_{m-1,\, m} = -k_b T (K_{m-1,\, m}) = -k_b T \left(\frac{k_{m-1,\, m}}{k_{m,\, m-1}} \right), \quad (2)$$

where $k_{m-1,m}$, $k_{m,m-1}$, and $K_{m-1,m}$ are box-to-box rate constants and equilibrium constant, respectively (see (17,19) for more details). BXD is very similar in spirit to US, which uses an additional parabolic potential to bias the dynamics instead of boxes. Unlike SMD, BXD trajectories are close to equilibrium as an equilibrium within each box is assumed and tested for.

Thus, SMD and BXD complement each other and allow to look at the dynamics of unfolding under two different regimes. SMD simulates fast and nonequilibrium unfolding, whereas BXD looks at slower unfolding describing nonequilibrium effects only by kinetics between the boxes (unpublished data). In this article, SMD and BXD are used to calculate the work profile and PMF of talin unfolding. Both the PMF itself and the differences between work profiles and PMFs calculated in two different ways provide useful information about the mechanism of protein unfolding.

BXD simulations were performed with the implicit solvent model, and SMD simulations were performed with the with explicit solvent model. To account for the difference in the description of water, we performed US with explicit water. In addition, to speed up SMD, coarse-grained SMD was performed. More technical details about BXD, SMD, US, and coarse-grained SMD can be found in the Materials and Methods.

MATERIALS AND METHODS

Structure preparation and analysis

Talin rod subdomains R9 and R3 were used as monomers, and subdomains R1–R2 as a tandem fragment were used in our simulations. The following structures from the Protein Data Bank (PDB) were used as initial models for the simulations: PDB: 2L7A for R3 (residues 796–909), PDB: 2KBB for R9 (residues 1657–1825), and PDB: 1SJ8 for R1–R2 dimer (residues 487–782). Analysis of the protein helicity was performed using DSSP (20) and includes α-, 3_{10}-, and π-helices.

BXD simulations

BXD simulations were performed using CHARMM (21) at the Taito supercomputer (CSC, Kajaani, Finland). The EEF1 implicit solvent model and CHARMM19 united-atom force field were used for the simulations together with the Langevin thermostat set to 300 K. The production BXD simulations were carried out with 0.5 nm window size, and the number of events was set to 1000 and 2000.

All-atom SMD simulations

SMD simulations were performed using Gromacs (22) at the Sisu supercomputer (CSC – IT Center for Science, Espoo, Finland). The CHARMM27 all-atom force field (23) and the explicit TIP3P water model (24) in 0.15 M KCl solution were used. The system preparation was performed using the same approach as described in our previous study (15), in which the energy minimization of the system is followed by three steps of equilibration of the system with harmonic potential restraints put on all-protein heavy atoms to allow the solution to take proper positions around the protein fold. Each system was then equilibrated up to 100 ns using NPT ensemble, in which V-rescale (25) and Berendsen (26) algorithms were used to maintain the system at 310 K and 1 bar, respectively. The temperature coupling was applied separately for the protein and solution parts. The snapshots at 5 ns for R9 and R3 monomers and at 15 ns for R1–R2 tandem were used as starting structures for the SMD simulations. The selection of the starting snapshots was based on the analysis of protein relaxation by monitoring the root mean-square deviation of the Cα atoms. In SMD simulations, the pressure coupling was switched off for the dimension of pulling, and the weak Berendsen algorithm was used for temperature coupling. The constant velocity pulling with 2 nm/ns speed was performed by applying the force to the Cα atom of the C-terminal protein residue and restraining the movements of the Cα atom of the N-terminal residue with harmonic potential. The spring constant was set to 1000 kJ/mol nm^2. The calculation of work done by pulling in SMD simulations was performed using PLUMED 2.5 (27).

PMF calculation using US

The US method was used to calculate the PMF for the unfolding of the talin rod subdomain R9. All sampling simulations were performed using Gromacs (22), and the final PMF was constructed using the weighted histogram analysis method as implemented in Gromacs. The starting structures for US simulations were taken from the SMD trajectory of R9 unfolding, in which 20 nm extension and 0.1 nm window size were used. An umbrella was set to 2000 kJ/mol nm^2. The Cα atom of the N-terminal residue was harmonically restrained with 2000 kJ/mol nm^2. Because the original SMD box size and hence the system was very big, the size of the box was adjusted for every single US simulation to reduce the computational resources and time needed. This process was automated and included generation of a system, energy minimization, and 1 ns equilibration of the system using harmonic position restraints at 2000 kJ/mol nm^2 put on all-protein heavy atoms. Thus, the size of each system lied between ~26,000 and 81,000 atoms. In total, 205 US simulations were performed, in which each was run was simulated for 30 ns. The last 20 ns of the US simulations were used to reconstruct the PMF profile.

Coarse-grained SMD

Coarse-grained simulations are performed using a structure-based model (28–31) with single amino acid resolution and interaction centers located on each amino acid's Cα atom. Peptide bonds connecting consecutive residues are simulated by a harmonic potential, with additional terms promoting native local chirality of the chain (32). Nonbonded interactions are given by a Lennard-Jones potential:

$$V_{LJ} = 4\varepsilon\left[\left(\frac{\sigma_{ij}}{r_{ij}}\right)^{12} - \left(\frac{\sigma_{ij}}{r_{ij}}\right)^{6}\right], \qquad (3)$$

with r_{ij} denoting the distance between amino acids i and j, and σ_{ij} chosen such that the minimum of the potential is located at the native distance of given pair of residues. The depth of the potential well is uniform for all interactions and was determined by comparison with protein unfolding experiments (29) to be 110 $\pm$ 10 pNÅ. Native nonbonded interactions are determined by the overlap criterion (33), i.e., for each heavy atom in the native structure, its van der Waals radius is determined and enlarged 1.24 times. If two heavy atoms from any pair of amino acids sequentially separated by at least two other residues overlap, they are said to form a native contact. For all remaining pairs of residues, their Lennard-Jones potential is set to be purely repulsive below $r_{ij} = 4$ Å.

The system is evolved in time using a fifth-order corrector-predictor algorithm according to Langevin dynamics, with dampening terms and thermal noise representing the implicit solvent. The dispersion of the noise is given by $\sqrt{2\gamma k_B T}$. Here, k_B is the Boltzmann constant, T is the temperature, and $\gamma = 2m/\tau$ is the damping coefficient, with m being the mass of the residue. The model is considered in an overdamped limit, with τ of the order of 1 ns (34,35), and the equations of motion are integrated with the time step of 0.005 τ. All simulations are performed at room temperature, i.e., $k_B T = 0.3\varepsilon$.

The unfolding is performed by attaching elastic springs (spring constant of $0.12\varepsilon/\text{Å}^2$) to both termini of the protein and moving one of the springs with a constant velocity v_p. In this study, unless stated otherwise in the text, the pulling velocity was set to 0.005 Å/τ.

Data availability

The simulation data, including coordinates, trajectories, and parameters, are available via the following address: https://doi.org/10.23729/e4805d6f-e85e-4712-8481-948dfc7c6f0e.

RESULTS

We combined BXD and SMD simulations to study the unfolding of talin rod subdomains, using these two techniques under different conditions (Fig. 1). In the BXD simulations, an implicit solvent model was used, making it possible to run a large number of replicas with minimal computational cost. For explicit solvent all-atom SMD simulations, the system size must be large enough (up to 60 nm) to accommodate the unfolded protein, making these simulations computationally demanding and limiting the possible number of replicas.

Before initiating the BXD production runs, a set of simulations was conducted to determine the parameters suitable

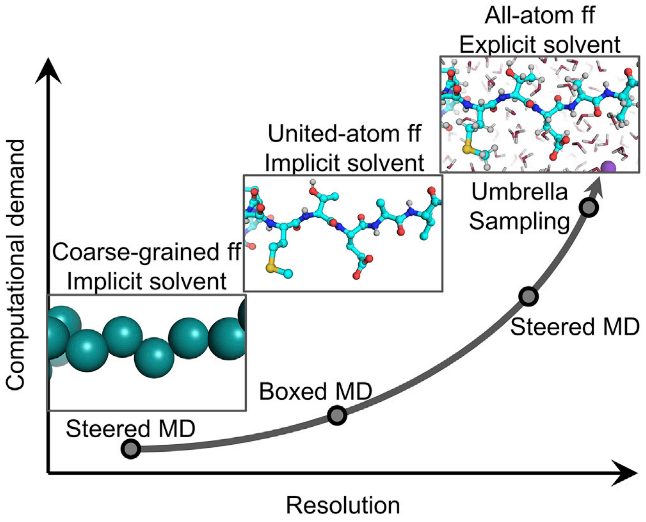

FIGURE 1 Nonequilibrium MD simulation techniques used for protein unfolding under mechanical load. MD simulations that use coarse-grained force field with the implicit solvent model are computationally the cheapest, whereas all-atom simulations with explicit solvent are the most computationally demanding but provide results at higher resolution. To see this figure in color, go online.

for the studied proteins, namely window size and number of collisions within a box (19). The window size of 0.5 nm was found suitable for talin rod subdomains, and the optimal number of events was 1000 and 2000. We conducted two sets of BXD simulations with different parameters, using both 1000 and 2000 events. We monitored the extension of the protein end-to-end distance as a function of time in the BXD simulations (Fig. S1). The trajectories generated with 2000 events for monomer bundles R9 and R3 and 1000 events for tandem R1–R2 were selected for further analysis, as they were the most reproducible.

Talin R9 and R3 unfolding in BXD

The talin rod subdomains R9 and R3 as monomer bundles were subjected to unfolding in BXD simulations using the end-to-end distance as a reaction coordinate. 11 independent replicas for each subdomain were generated. To determine the nature of the bundle unfolding and to estimate the likelihood of the partially unfolded structure, including the three-helix intermediate (15), we analyzed the PMF for complete bundle unfolding. The PMF profiles for R9 suggest that the whole unfolding occurs in two events that correspond to the transition from a folded five-helix state to the three-helix intermediate and then to a completely unfolded structure (zero-helix state) (Fig. 2 *a*). Similarly, R3 has one unfolding event corresponding to the breaking of the three-helix state (Fig. 2 *b*). The PMF values always increase during the bundle extension, showing a higher local increase associated with the breaking of the five- or three-helix state, which suggests that the folded bundle is the most favorable conformation.

Analysis of the evolution of the protein secondary structure during the simulation (Fig. S2) showed refolding of α-helices after breaking the five- or three-helix state (Fig. 2, *c* and *d*). The α-helical secondary structure breaks during unfolding; however, once the five- or three-helix state is collapsed and the tension applied to the structure is released, the secondary structure of the dissociated helices refolds. The observed refolding was most effective for partially unfolded helices.

The generated replicas have a stochastic nature, having slightly different PMF values and the unfolding events having different positions. We selected 2 (out of 11) representative replicas for both R9 and R3 (*highlighted* in Fig. 2), which show lower and higher PMF values in the unfolding profiles. Analysis of the unfolding pathways showed that the difference in the PMF values was caused by different unfolding mechanisms. The replicas showing lower PMF represent a mechanism in which the α-helices are detached from the rest of the protein with minimal helix uncoiling and thus preserve more helicity during unfolding. In contrast, the replicas showing higher PMF represent the unfolding mechanism in which the helices unfold before dissociation from the rest of the protein, which results in the increase of PMF values (Fig. 2, *e* and *f*).

Talin R9 and R3 unfolding in all-atom SMD

We then subjected the R9 and R3 subdomains to mechanical unfolding in all-atom SMD. To compare the BXD and SMD results, we analyzed the work done by pulling in SMD. The work profiles (Fig. 3, *a* and *b*) are very similar to the PMF profiles from BXD, representing an increase of the values during the bundle unfolding with two unfolding events for R9 and one for R3. The magnitudes of the PMF and the work are not useful as the profiles represent qualitative results only; however, the work values from SMD are much higher compared with PMF values from BXD. Similarly, significant refolding of the secondary structure after breaking the five- or three-helix structure was not observed (Fig. 3, *c* and *d*; Fig. S3). This is due to a higher unfolding speed in SMD than in BXD caused by the application of force with constant velocity at 2 nm/ns. During the unfolding, the α-helices are immediately uncoiled in SMD, and thus, the SMD results are more similar to BXD replicas with a higher magnitude of PMF values. The structure snapshots captured from representative replicas (one out of five) are shown in Fig. 3, *f* and *g*.

Both the work and higher PMF values for R9 suggest that R9 is much stronger than R3 in the response to mechanical load.

PMF profile for talin R9 unfolding obtained from US supports BXD results

To obtain a PMF profile for the R9 unfolding pathway provided by the all-atom SMD and to compare this with the

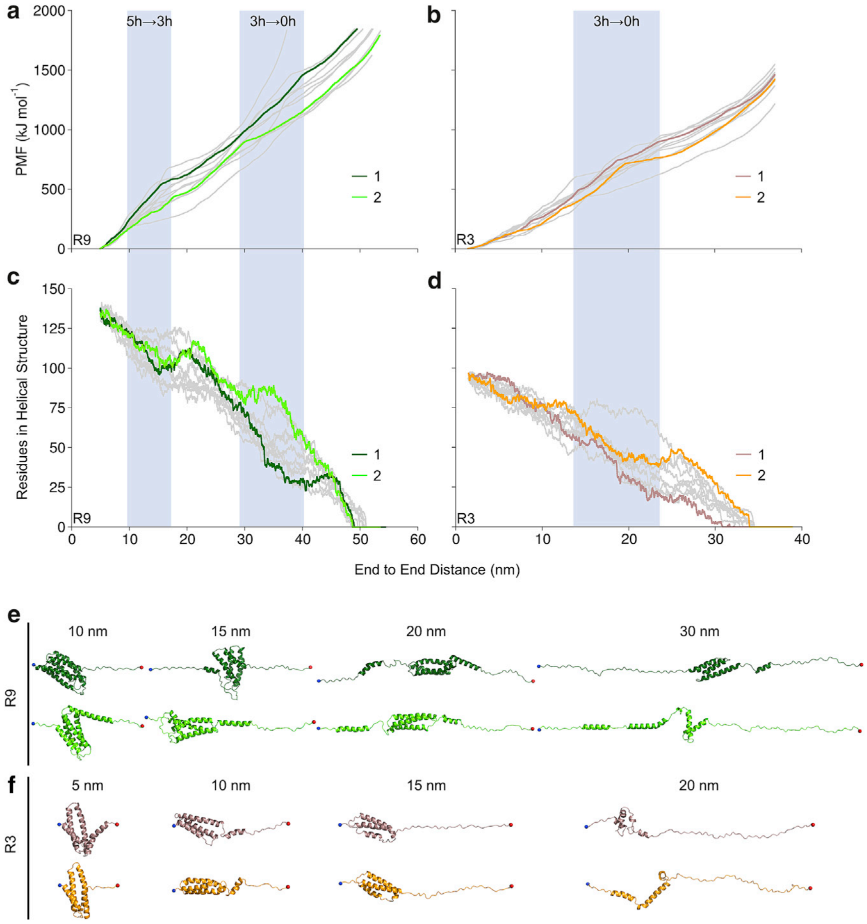

FIGURE 2 Unfolding of talin rod R9 and R3 in BXD simulations. (*a* and *b*) PMF of unfolding and secondary structure helicity (*c* and *d*) for R9 and R3, respectively. Highlighted with (*a* and *c*) dark green and light green for R9 and (*b* and *d*) with brown and orange for R3 are 2 representative replicas out of 11 (the remaining 9 replicas are shown in *gray*) that were generated in BXD simulations. Unfolding events associated with the transition of the folded five-helix bundle to the three-helix intermediate and then to the completely unfolded structure (zero-helix state) for R9 and three-helix to the zero-helix state for R3 are highlighted with blue background. Shown are structure snapshots of the representative replicas captured at (*e*) 10, 15, 20, and 30 nm for R9 and (*f*) at 5, 10, 15, and 20 nm for R3. The protein helicity was calculated using DSSP. To see this figure in color, go online.

PMF from BXD, we employed US. Because the reaction coordinate for complete R9 unfolding is very long (~50 nm), we used the first 20 nm of R9 extension, which represents unfolding to the three-helix intermediate. The structure snapshots captured at every 0.1 nm from the SMD trajectory were used as starting conformations for the US windows. The PMF profile from US was in very good agreement with that obtained by BXD and very similar to BXD replicas with a higher magnitude of PMF values (Fig. 3 *e*). Similarly to BXD, the PMF values increase during the unfolding of the five-helix R9 to the three-helix intermediate, showing a local higher increase associated with the breaking of the five-helix structure. Importantly, in US calculations, we employed the explicit solvent model like in SMD. The fact that the US PMF with explicit water model is below that of SMD suggests that high pulling speed alters the unfolding pathway, but this requires future investigation.

Talin R9 and R3 unfolding in coarse-grained SMD

To investigate how the talin rod subdomains R9 and R3 unfold in SMD when a much lower pulling speed is used along with a large number of repetitions, we employed amino acid-level coarse-grained simulations. A constant velocity pulling at 0.05 nm/ns was applied to stretch the bundles, and 20 parallel replicas for each were generated. We analyzed the unfolding force profiles, and the results are similar to our all-atom SMD findings (Fig. 4, *a* and *b*). In coarse-grained SMD, R9 has two unfolding events, and R3 has one major unfolding event (Fig. 4, *c* and *d*). However, the analysis of unfolding pathways showed that in contrast to all-atom SMD, the first unfolding event for R9 represents the collapse of the five-helix state and dissociation of H1 from the rest of the protein, followed by relaxation and subsequently dissociation of H5 (Fig. 4 *e*). The second unfolding event for R9, similarly to all-atom SMD, represents the collapse of the three-helix intermediate, which was formed by H2–H4 in 12 out of 20 replicas. Unfolding of R3 is in good agreement with all-atom SMD, in which the main unfolding event corresponds to the breaking of the three-helix state. However, the transition from a four-helix to three-helix structure in the coarse-grained SMD showed a significant peak in the unfolding force. The four-helix R3 unfolds to the three-helix intermediate by dissociation of either H1 or H4 from the rest of the protein (Fig. 4 *f*); thus, the three-helix state is formed by

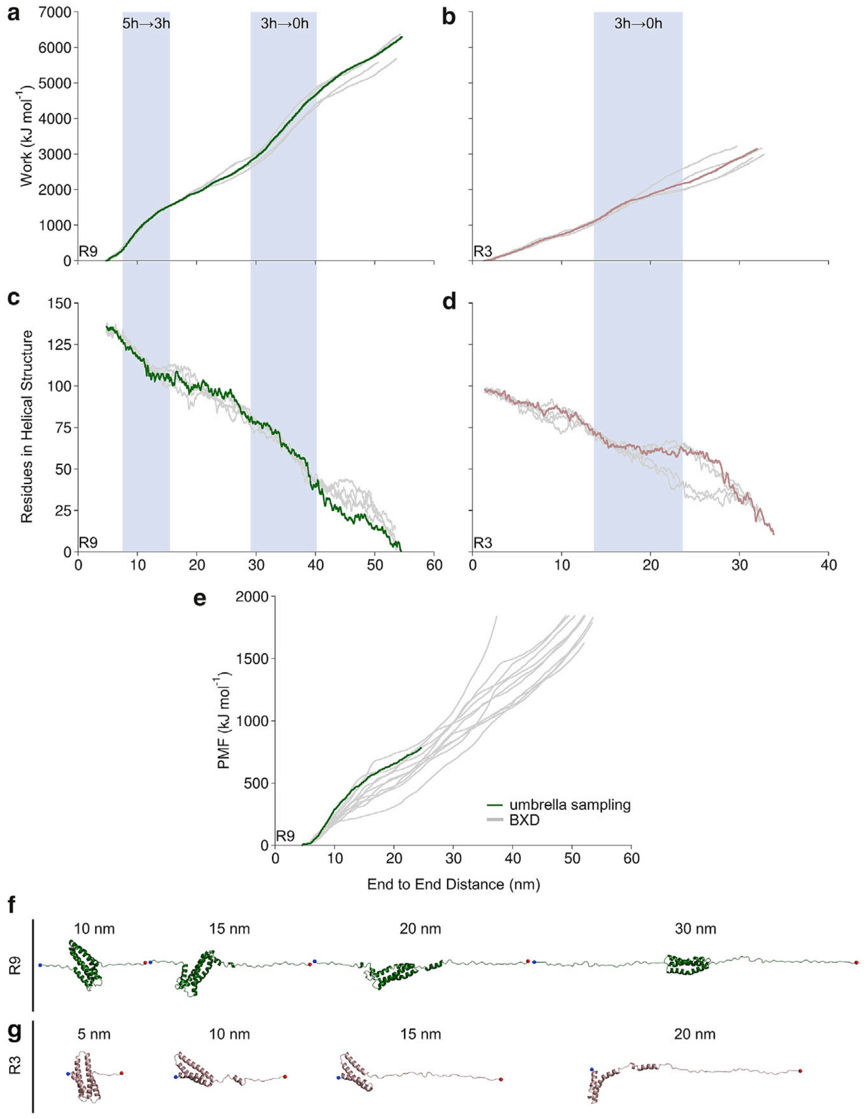

FIGURE 3 Unfolding of talin rod R9 and R3 in all-atom SMD simulations. (*a* and *b*) Work done by pulling and secondary structure helicity (*c* and *d*) for R9 and R3, respectively. Highlighted with (*a* and *c*) green for R9 and (*b* and *d*) with brown for R3 is a representative replica out of five (the remaining four replicas are shown in *gray*) that were generated in SMD simulations. Unfolding events associated with the transition of the folded five-helix bundle to the three-helix intermediate and then to the completely unfolded structure (zero-helix state) for R9 and three-helix to zero-helix state for R3 are highlighted with a blue background. (*e*) PMF of R9 unfolding using US (*green*) and BXD (*gray*). Shown are the structure snapshots of the representative replica captured at (*f*) 10, 15, 20, and 30 nm for R9 and (*g*) at 5, 10, 15, and 20 nm for R3. The protein helicity was calculated using DSSP. To see this figure in color, go online.

either H1-H3 or H2-H4. The breaking of the three-helix structure causes the highest peak in the unfolding force, suggesting that the three-helix intermediate is the strongest conformation of the R3.

Talin rod R1–R2 tandem domain unfolding in BXD

We next studied how the talin rod R1–R2 tandem unfolds under mechanical load by employing BXD simulations. Similarly to the BXD simulations on R9 and R3 monomers, 11 independent replicas were generated. The talin rod subdomains R1 and R2 interact via an extensive hydrophobic interface (36); therefore, the unfolding mechanism for the tandem is more complex than for the monomer. The PMF increased during the protein extension in which two major unfolding events can be identified, belonging to the unfolding of the R1 and R2 bundles. The PMF profiles for R1–R2 also have a stochastic nature, and we selected two replicas that represent lower and higher PMF (Fig. 5). Analysis of the protein secondary structure evolution during unfolding (Fig. S4) showed recoiling of the α-helices after the unfolding events, and the replica with the lower PMF preserving more helicity during unfolding. Another significant factor influencing the PMF is whether the R1–R2 interaction interface breaks, causing dissociation before unfolding. The representative replicas 1 and 2 in Fig. 5 have an approximately equal number of residues in helical structure until ~40 nm of end-to-end distance; however, the PMF is lower for replica 2 because of dissociation of R1 and R2 subdomains at the beginning of the simulation. In replica 1, R1 and R2 did not dissociate before complete unfolding. After ~40 nm of end-to-end distance, the number of residues in helical structure increases for replica 2 because of complete unfolding of R2 and refolding of the secondary structure.

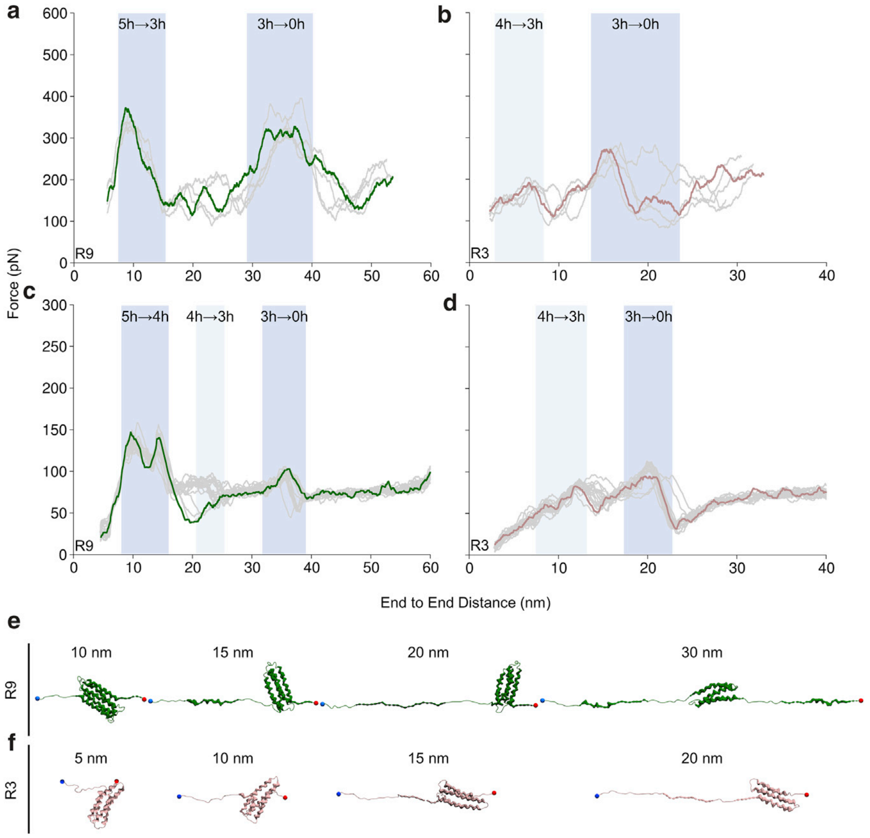

FIGURE 4 Unfolding of talin rod R9 and R3 in all-atom and coarse-grained SMD simulations. Unfolding force profiles are shown as function of the protein end-to-end distance in (*a* and *b*) all-atom and (*c* and *d*) coarse-grained SMD for R9 and R3, respectively. Unfolding events associated with the collapse of the five-helix bundle for R9 and the three-helix intermediate for both R9 and R3 are highlighted with a dark blue background, and the transition of the four-helix to the three-helix structure is highlighted with a light blue background. Shown are structure snapshots of the representative coarse-grained SMD replicas captured at (*e*) 10, 15, 20, and 30 nm for R9 and (*f*) at 5, 10, 15, and 20 nm for R3. To see this figure in color, go online.

In the BXD simulations, R1 and R2 did not dissociate before the complete unfolding in 7 out of 11 replicas. The four-helix R2 is mechanically weaker than the five-helix R1 (15) and unfolded first in the tandem in 8 out 11 replicas. Both R1 and R2 subdomains unfolded through three-helix states, formed by H2–H3 in R1 and H1–H3 in R2.

R1–R2 tandem unfolding in all-atom SMD

The R1–R2 fragment of the talin rod was then subjected to mechanical unfolding in all-atom SMD. Because of the large system size (754,087 atoms), only three replicas were generated. Analysis of the work done by pulling in SMD and the unfolding pathways (Fig. 6) showed two major unfolding events. In all three replicas, the first event is complete unfolding of R2 (~45 nm of the end-to-end distance), and the second unfolding event is the complete unfolding of R1 (~78 nm). This is due to the fact that R2 is mechanically weaker than R1 (15); however, the pulling was applied to the C-terminus of the R1–R2 fragment, which facilitates unfolding of the structure located closer to the pulling point, R2. The hydrophobic interface between R1 and R2 breaks before R2 unfolding in all three replicas. Similarly to BXD, both R1 and R2 bundles unfolded through three-helix states, formed by H2–H3 in R1 and H1–H3 in R2. Analysis of the secondary structure evolution (DSSP analysis) is shown in Fig. S5.

DISCUSSION

Our previous studies have shown that the talin rod subdomains have different responses to mechanical load because they possess varying mechanical stability (12). The individual subdomains unfold through a stable three-helix intermediate, which is the most stable state for four-helix bundles and the second-most stable state for five-helix rod subdomains (15). The mechanical unfolding was studied using computational and experimental tools assessing the unfolding force patterns; however, the PMF for the unfolding was not investigated. Here, we take advantage of the BXD technique to determine the unfolding PMF profiles for the individual talin rod subdomains and cross-validate them with US. We demonstrate how the combination of BXD, SMD, and US techniques can help to build a comprehensive understanding about protein behavior under mechanical load and provide a comparative analysis of the BXD, SMD, and US techniques using different levels of molecular resolution: from an all-atom force field together with an explicit water model to a coarse-grained force field with implicit solvent.

The BXD method was previously used to study the unfolding of α-helical protein L, β-strand titin I27, and α–β IM9, which are smaller than the talin rod subdomains (17,37). Here, we demonstrate that BXD can be applied for bigger α-helical proteins (R9 169 residues, R3 114 residues, and R1–R2 296 residues), which are known to be

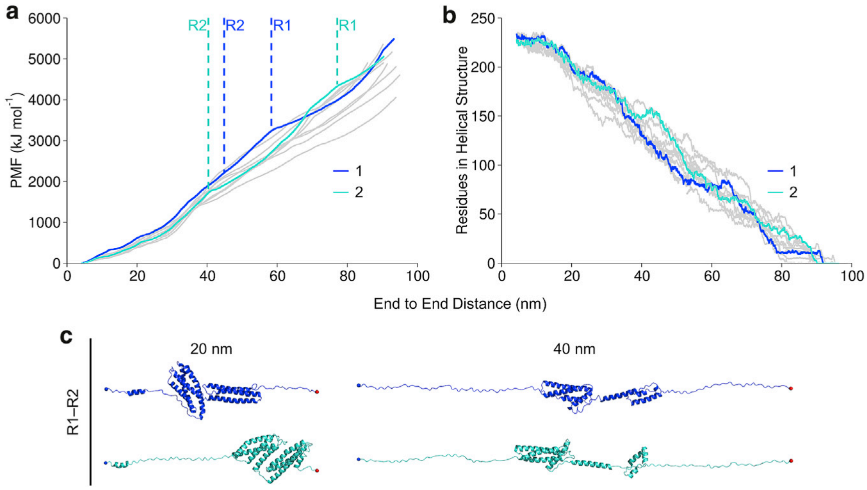

FIGURE 5 Unfolding of the talin rod R1–R2 fragment in BXD simulations. (*a*) PMF of unfolding and secondary structure helicity (*b*) for the R1–R2 fragment. Highlighted with blue and cyan (1,2) are 2 representative replicas out of 11 (remaining 9 replicas are shown in *gray*) that were generated in BXD simulations. (*c*) Structure snapshots of the representative replicas that were captured at 20 and 40 nm. The protein helicity was calculated using DSSP. Unfolding events for R1 and R2 are shown with dashed lines. To see this figure in color, go online.

mechanically softer that β-strand fold and therefore more challenging to manage using both theoretical and experimental tools (38).

The BXD simulations were conducted using implicit solvent, dramatically reducing the computational cost and time needed to perform the simulations compared to an all-atom system model. Hence, a higher number of replicas was generated, and the unfolding was carried out much slower than in all-atom SMD. Altogether, this brings BXD closer to the experimental methods used to study protein unfolding, such as single molecule AFM or magnetic tweezers. Comparing the PMF for R9 from BXD to unfolding patterns obtained by AFM (12), one can clearly see a similar stochasticity of positions of the unfolding events. The first unfolding event is less stochastic, whereas the second unfolding event, which involves the breaking of the three-helix state, is more stochastic and takes place in a range from ~28 to 40 nm of end-to-end distance in BXD. In all-atom SMD, the positions of the unfolding events are less stochastic than in BXD. The stochasticity in BXD can be explained by the fact that the use of the implicit solvent increases the speed of the sampling of conformational space due to a reduction of the solvent viscosity (39).

Based on our experience of using coarse-grained SMD, BXD, and all-atom SMD simulations, we propose a workflow of combining these techniques (Fig. 7). The main advantage of coarse-grained SMD using implicit solvent is the possibility to unfold large protein constructs with a high number of repetitions. It allows estimation of the unfolding pathway and the prediction of the unfolding force profile. This makes coarse-grained SMD an excellent tool for screening of either large multidomain proteins or a large number of proteins. Based on coarse-grained SMD data, a limited number of the most interesting proteins/domains could be selected for more detailed analysis with BXD and all-atom SMD. BXD simulations were used with

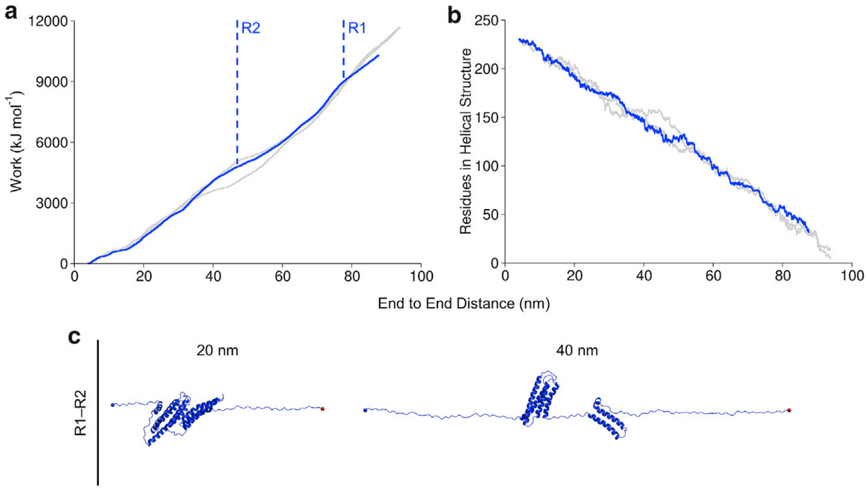

FIGURE 6 Unfolding of talin rod R1–R2 fragment in all-atom SMD simulations. (*a*) Work done by pulling and secondary structure helicity (*b*) for the R1–R2 fragment. Highlighted with blue is a representative replica out of three (remaining two replicas are shown in *gray*) that were generated in SMD simulations. (*c*) Structure snapshots of the representative replicas that were captured at 20 and 40 nm. The protein helicity was calculated using DSSP. Unfolding events for R1 and R2 are shown with dashed lines. To see this figure in color, go online.

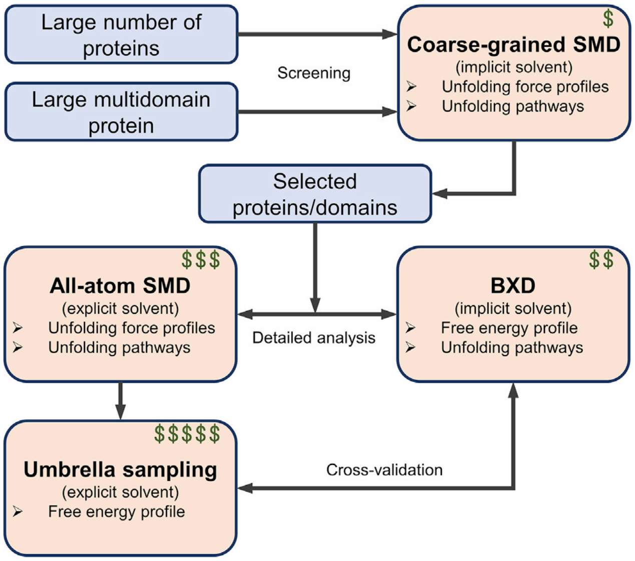

FIGURE 7 Schematic workflow of combining coarse-grained SMD, BXD, all-atom SMD, and US for the study of protein unfolding under mechanical load. The dollar sign represents computational resources needed to conduct the simulations, with coarse-grained simulations being the cheapest and US the most computationally expensive. To see this figure in color, go online.

united-atom force field and implicit solvent; therefore, a high number of replicas for a single domain protein could be generated. The strongest merit of the BXD approach is the possibility to estimate the PMF of the unfolding process. However, the BXD setup requires the finding of appropriate parameters for protein unfolding, namely box size and number of events, to allow the protein extension with good sampling and is thus more appropriate for single domain protein constructs. Finally, all-atom SMD is a flexible and powerful tool for protein unfolding and is very good in combination with BXD for cross-validation of the experiments. Using an explicit water model for protein unfolding enables detailed understanding of the unfolding mechanisms but makes the approach computationally expensive because of the large system size. Therefore, protein size, number of replicas, and extension distance are factors to be considered. Protein unfolding in SMD could be further analyzed with US to construct the PMF; however, because of the very long ($\sim$50 nm) reaction coordinate, the number of US simulations required to construct the PMF are too expensive computationally.

The unfolding free energy landscapes provided in the current study show that during unfolding, the PMF increases from the very beginning until complete bundle unfolding, with no local energy minimum. This suggests that the folded state is the most favorable conformation of the talin subdomains, and the intermediate states can exist under mechanical load only. This finding implicates that in the absence of force or reduction of mechanical load, partially unfolded talin rod subdomains should spontaneously refold; however, the refolding mechanism requires further investigation.

Previous studies have shown that mechanical stretching of talin exposes binding sites for vinculin (6,9,10). Binding of vinculin to talin inhibits talin in an unfolded conformation, and under higher mechanical load (>25 pN), vinculin dissociates from talin (14,40). This suggests that binding of vinculin to talin requires the folded conformation of α-helices, and at large forces, the α-helical secondary structure becomes unstable, leading to dissociation of vinculin. A recent study suggests that vinculin binding can contribute to talin helix refolding (41). All-atom SMD suggests an unfolding mechanism in which the α-helical secondary structure permanently breaks during subdomain unfolding (Fig. 3, *c* and *d*), and no significant refolding was observed because of the high pulling speed applied to stretch the protein. In contrast to all-atom SMD, in BXD, the protein α-helical secondary structure was not completely unfolded during the breaking of the bundle tertiary structure, especially in replicas with lower unfolding PMF, and significant refolding of individual α-helices was observed (Fig. 2, *c* and *d*).

Collectively, our BXD and SMD simulations suggest a model of unfolding of the talin rod subdomains, explaining the mechanism of structure breaking and refolding of α-helices, which is crucial for vinculin binding (Fig. S6). Under mechanical load, either the completely folded bundle or three-helix intermediate breaks, and during this process, the α-helical secondary structure unfolds but not completely. The α-helices preserve some helical structure after dissociation from the rest of the protein and then rapidly refold because of low mechanical load and, consequently, the low speed of unfolding. After the refolding of the secondary structure, vinculin-binding helices become capable of complexation with vinculin. If the mechanical load on the talin rod is reduced, the three-helix intermediates and completely unfolded bundles refold their tertiary structure, unless they are "locked" into an unfolded conformation by bound vinculin (14). This model assumes that the unfolding occurs with low mechanical load ($\sim$40 pN) in the microsecond-to-minute time frame, typical for in vivo conditions and currently not accessible by simulations.

CONCLUSION

We have presented an analysis of mechanical unfolding of talin rod subdomains R9 and R3 and R1–R2 tandem using BXD and SMD simulations at a different detail level. We demonstrated that BXD with implicit solvent is a powerful tool for the calculation of PMF profiles for protein unfolding at low computational cost. Coarse-grained SMD is very good for screening of a large number of proteins, whereas all-atoms simulations provide the highest detail level.

PMF plots obtained from BXD revealed that the three-helix intermediate in talin rod subdomains is stabilized by force during mechanical unfolding, and US confirmed these

results. The BXD simulations revealed that the α-helical secondary structure refolds after the breaking of the bundle tertiary structure, which is probably crucial for the recruitment of vinculin to partially or completely unfolded talin rod subdomains. We propose a general workflow applicable for the study of the mechanical response of proteins with different MD methods.

SUPPORTING MATERIAL

Supporting Material can be found online at https://doi.org/10.1016/j.bpj.2020.07.030.

AUTHOR CONTRIBUTIONS

V.V.M. built up the concept of the study, designed the research, conducted BXD, all-atom SMD, and US simulations, analyzed the results, and wrote the manuscript, supervised by V.P.H. M.S. performed coarse-grained SMD simulations, and analysis was supervised by M.C. J.J.B. provided significant inputs to BXD experiments, supervised by D.V.S.

ACKNOWLEDGMENTS

We acknowledge the CSC – IT Center for Science for computational resources. We thank Giovanni Bussi (International School for Advanced Studies) and Alessandro Laio (International School for Advanced Studies) for the valuable discussion of this work.

This work was supported by the Academy of Finland grant 290506 to V.P.H. V.V.M. was supported by Academy of Finland grant 323021. M.C. has received support from the National Science Centre of Poland, under grant 2018/31/B/NZ1/00047. D.V.S. acknowledges the support of Engineering and Physical Sciences Research Council, grant EP/P021123/1.

REFERENCES

1. Hu, X., F. M. Margadant, …, M. P. Sheetz. 2017. Molecular stretching modulates mechanosensing pathways. *Protein Sci.* 26:1337–1351.
2. Seifert, C., and F. Gräter. 2013. Protein mechanics: how force regulates molecular function. *Biochim. Biophys. Acta.* 1830:4762–4768.
3. Galbraith, C. G., K. M. Yamada, and M. P. Sheetz. 2002. The relationship between force and focal complex development. *J. Cell Biol.* 159:695–705.
4. Wehrle-Haller, B. 2012. Assembly and disassembly of cell matrix adhesions. *Curr. Opin. Cell Biol.* 24:569–581.
5. Calderwood, D. A., I. D. Campbell, and D. R. Critchley. 2013. Talins and kindlins: partners in integrin-mediated adhesion. *Nat. Rev. Mol. Cell Biol.* 14:503–517.
6. Goult, B. T., T. Zacharchenko, …, I. L. Barsukov. 2013. RIAM and vinculin binding to talin are mutually exclusive and regulate adhesion assembly and turnover. *J. Biol. Chem.* 288:8238–8249.
7. Li, G., X. Du, …, X. Qian. 2011. Full activity of the deleted in liver cancer 1 (DLC1) tumor suppressor depends on an LD-like motif that binds talin and focal adhesion kinase (FAK). *Proc. Natl. Acad. Sci. USA.* 108:17129–17134.
8. Bouchet, B. P., R. E. Gough, …, A. Akhmanova. 2016. Talin-KANK1 interaction controls the recruitment of cortical microtubule stabilizing complexes to focal adhesions. *Elife.* 5:e18124.
9. del Rio, A., R. Perez-Jimenez, …, M. P. Sheetz. 2009. Stretching single talin rod molecules activates vinculin binding. *Science.* 323:638–641.
10. Hytönen, V. P., and V. Vogel. 2008. How force might activate talin's vinculin binding sites: SMD reveals a structural mechanism. *PLoS Comput. Biol.* 4:e24.
11. Kluger, C., L. Braun, …, V. Vogel. 2020. Different vinculin binding sites use the same mechanism to regulate directional force transduction. *Biophys. J.* 118:1344–1356.
12. Haining, A. W. M., M. von Essen, …, A. Del Río Hernández. 2016. All subdomains of the talin rod are mechanically vulnerable and may contribute to cellular mechanosensing. *ACS Nano.* 10:6648–6658.
13. Fillingham, I., A. R. Gingras, …, I. L. Barsukov. 2005. A vinculin binding domain from the talin rod unfolds to form a complex with the vinculin head. *Structure.* 13:65–74.
14. Yao, M., B. T. Goult, …, J. Yan. 2014. Mechanical activation of vinculin binding to talin locks talin in an unfolded conformation. *Sci. Rep.* 4:4610.
15. Mykuliak, V. V., A. W. M. Haining, …, V. P. Hytönen. 2018. Mechanical unfolding reveals stable 3-helix intermediates in talin and α-catenin. *PLoS Comput. Biol.* 14:e1006126.
16. Sumbul, F., and F. Rico. 2019. Single-molecule force spectroscopy: experiments, analysis, and simulations. *In* Atomic Force Microscopy: Methods and Protocols. N. C. Santos and F. A. Carvalho, eds. Springer, pp. 163–189.
17. Booth, J., S. Vazquez, …, D. V. Shalashilin. 2014. Recent applications of boxed molecular dynamics: a simple multiscale technique for atomistic simulations. *Philos. Trans. A Math. Phys. Eng. Sci.* 372:20130384.
18. Genchev, G. Z., M. Källberg, …, H. Lu. 2009. Mechanical signaling on the single protein level studied using steered molecular dynamics. *Cell Biochem. Biophys.* 55:141–152.
19. Glowacki, D. R., E. Paci, and D. V. Shalashilin. 2009. Boxed molecular dynamics: a simple and general technique for accelerating rare event kinetics and mapping free energy in large molecular systems. *J. Phys. Chem. B.* 113:16603–16611.
20. Kabsch, W., and C. Sander. 1983. Dictionary of protein secondary structure: pattern recognition of hydrogen-bonded and geometrical features. *Biopolymers.* 22:2577–2637.
21. Brooks, B. R., C. L. Brooks, III, …, M. Karplus. 2009. CHARMM: the biomolecular simulation program. *J. Comput. Chem.* 30:1545–1614.
22. Abraham, M. J., T. Murtola, …, E. Lindah. 2015. Gromacs: high performance molecular simulations through multi-level parallelism from laptops to supercomputers. *SoftwareX.* 1–2:19–25.
23. MacKerell, A. D., D. Bashford, …, M. Karplus. 1998. All-atom empirical potential for molecular modeling and dynamics studies of proteins. *J. Phys. Chem. B.* 102:3586–3616.
24. Jorgensen, W. L., and J. D. Madura. 1983. Quantum and statistical mechanical studies of liquids. 25. Solvation and conformation of methanol in water. *J. Am. Chem. Soc.* 105:1407–1413.
25. Bussi, G., D. Donadio, and M. Parrinello. 2007. Canonical sampling through velocity rescaling. *J. Chem. Phys.* 126:014101.
26. Berendsen, H. J. C., J. P. M. Postma, …, J. R. Haak. 1984. Molecular dynamics with coupling to an external bath. *J. Chem. Phys.* 81:3684–3690.
27. Tribello, G. A., M. Bonomi, …, G. Bussi. 2014. PLUMED 2: new feathers for an old bird. *Comput. Phys. Commun.* 185:604–613.
28. Sułkowska, J. I., and M. Cieplak. 2007. Mechanical stretching of proteins—a theoretical survey of the Protein Data Bank. *J. Phys. Condens. Matter.* 19:283201.
29. Sikora, M., J. I. Sułkowska, and M. Cieplak. 2009. Mechanical strength of 17,134 model proteins and cysteine slipknots. *PLoS Comput. Biol.* 5:e1000547.
30. Sikora, M., and M. Cieplak. 2011. Mechanical stability of multidomain proteins and novel mechanical clamps. *Proteins.* 79:1786–1799.
31. Różycki, B., Ł. Mioduszewski, and M. Cieplak. 2014. Unbinding and unfolding of adhesion protein complexes through stretching: interplay between shear and tensile mechanical clamps. *Proteins.* 82:3144–3153.

32. Kwiecińska, J. I., and M. Cieplak. 2005. Chirality and protein folding. *J. Phys. Condens. Matter.* 17:S1565–S1580.

33. Wołek, K., À. Gómez-Sicilia, and M. Cieplak. 2015. Determination of contact maps in proteins: a combination of structural and chemical approaches. *J. Chem. Phys.* 143:243105.

34. Veitshans, T., D. Klimov, and D. Thirumalai. 1997. Protein folding kinetics: timescales, pathways and energy landscapes in terms of sequence-dependent properties. *Fold. Des.* 2:1–22.

35. Szymczak, P., and M. Cieplak. 2006. Stretching of proteins in a uniform flow. *J. Chem. Phys.* 125:164903.

36. Papagrigoriou, E., A. R. Gingras, …, J. Emsley. 2004. Activation of a vinculin-binding site in the talin rod involves rearrangement of a five-helix bundle. *EMBO J.* 23:2942–2951.

37. Booth, J. J., and D. V. Shalashilin. 2016. Fully atomistic simulations of protein unfolding in low speed atomic force microscope and force clamp experiments with the help of boxed molecular dynamics. *J. Phys. Chem. B.* 120:700–708.

38. Carrion-Vazquez, M., A. F. Oberhauser, …, J. M. Fernandez. 2000. Mechanical design of proteins studied by single-molecule force spectroscopy and protein engineering. *Prog. Biophys. Mol. Biol.* 74:63–91.

39. Anandakrishnan, R., A. Drozdetski, …, A. V. Onufriev. 2015. Speed of conformational change: comparing explicit and implicit solvent molecular dynamics simulations. *Biophys. J.* 108:1153–1164.

40. Ciobanasu, C., B. Faivre, and C. Le Clainche. 2014. Actomyosin-dependent formation of the mechanosensitive talin-vinculin complex reinforces actin anchoring. *Nat. Commun.* 5:3095.

41. Tapia-Rojo, R., A. Alonso-Caballero, and J. M. Fernandez. 2020. Direct observation of a coil-to-helix contraction triggered by vinculin binding to talin. *Sci. Adv.* 6:eaaz4707.

Biophysical Journal, Volume *119*

Supplemental Information

Mechanical Unfolding of Proteins—A Comparative Nonequilibrium Molecular Dynamics Study

Vasyl V. Mykuliak, Mateusz Sikora, Jonathan J. Booth, Marek Cieplak, Dmitrii V. Shalashilin, and Vesa P. Hytönen

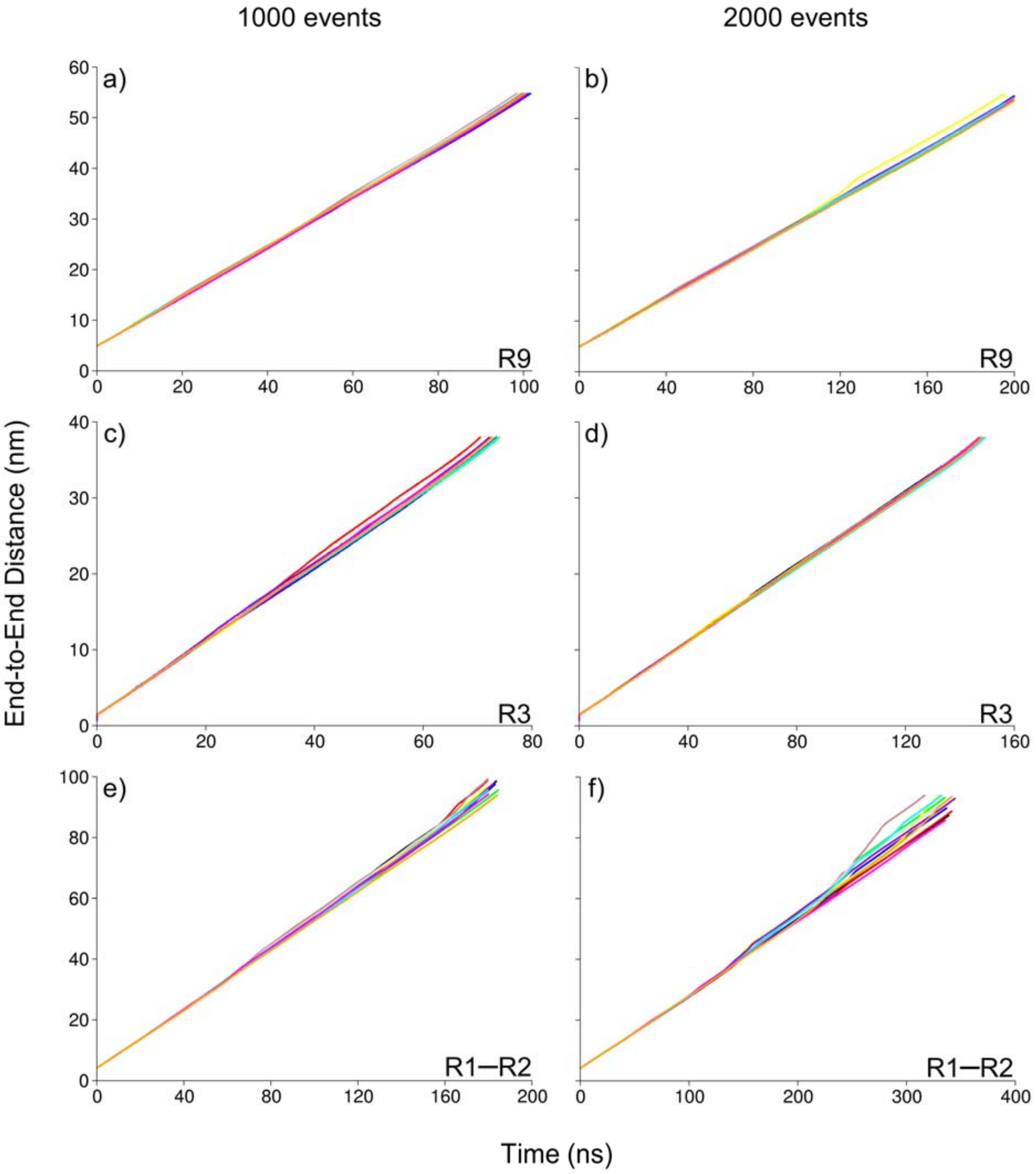

Figure S1. End-to-end distance as a function of time for the talin rod monomers (a & b) R9 and (c & d) R3, and (e & f) the tandem fragment R1–R2 during unfolding in BXD simulations, using (a, c & e) 1000 events and (b, d & f) 2000 events.

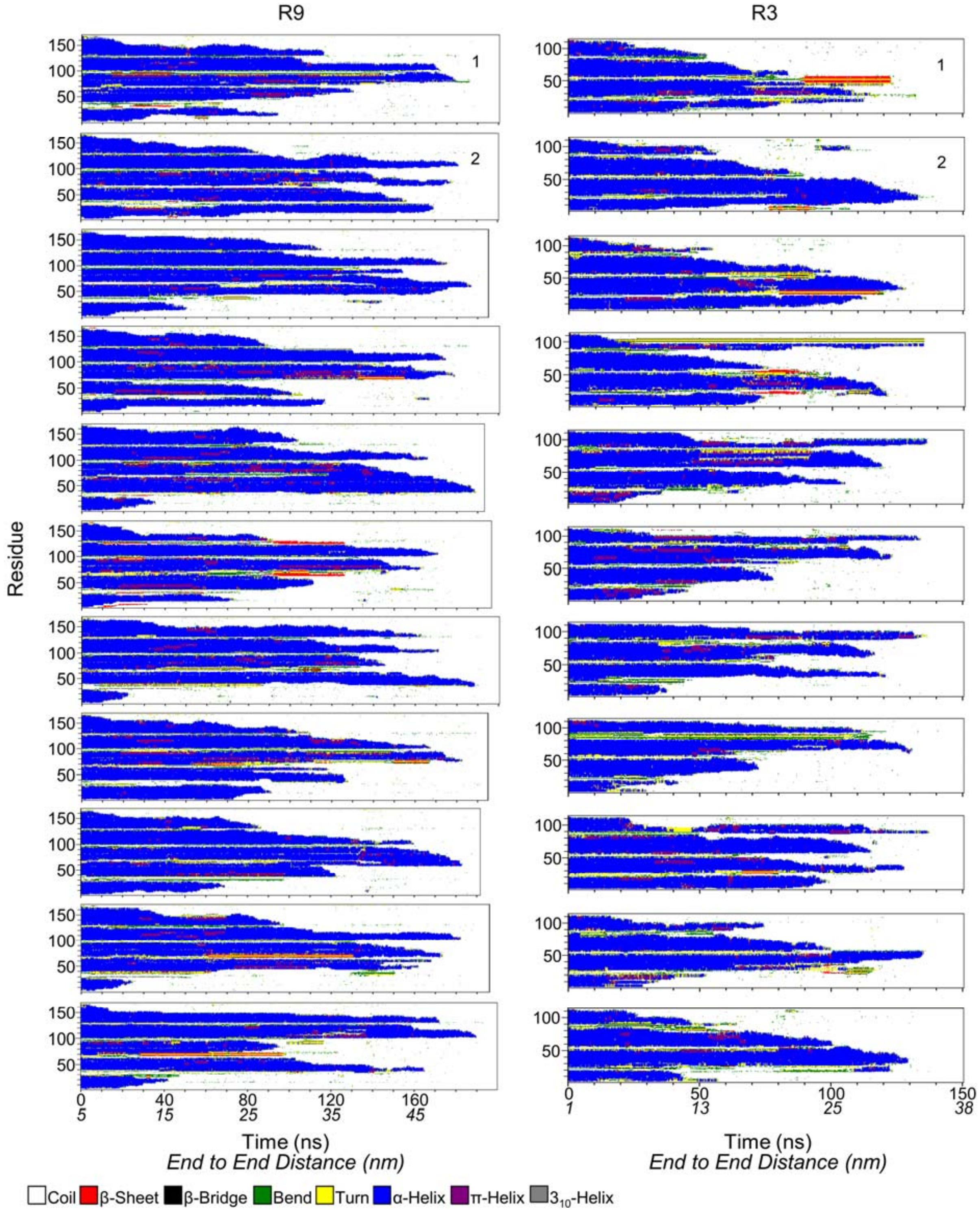

Figure S2. Secondary structure analysis of talin rod (left) R9 and (right) R3 during unfolding in BXD simulations. The analysis was performed using DSSP, for all eleven replicas.

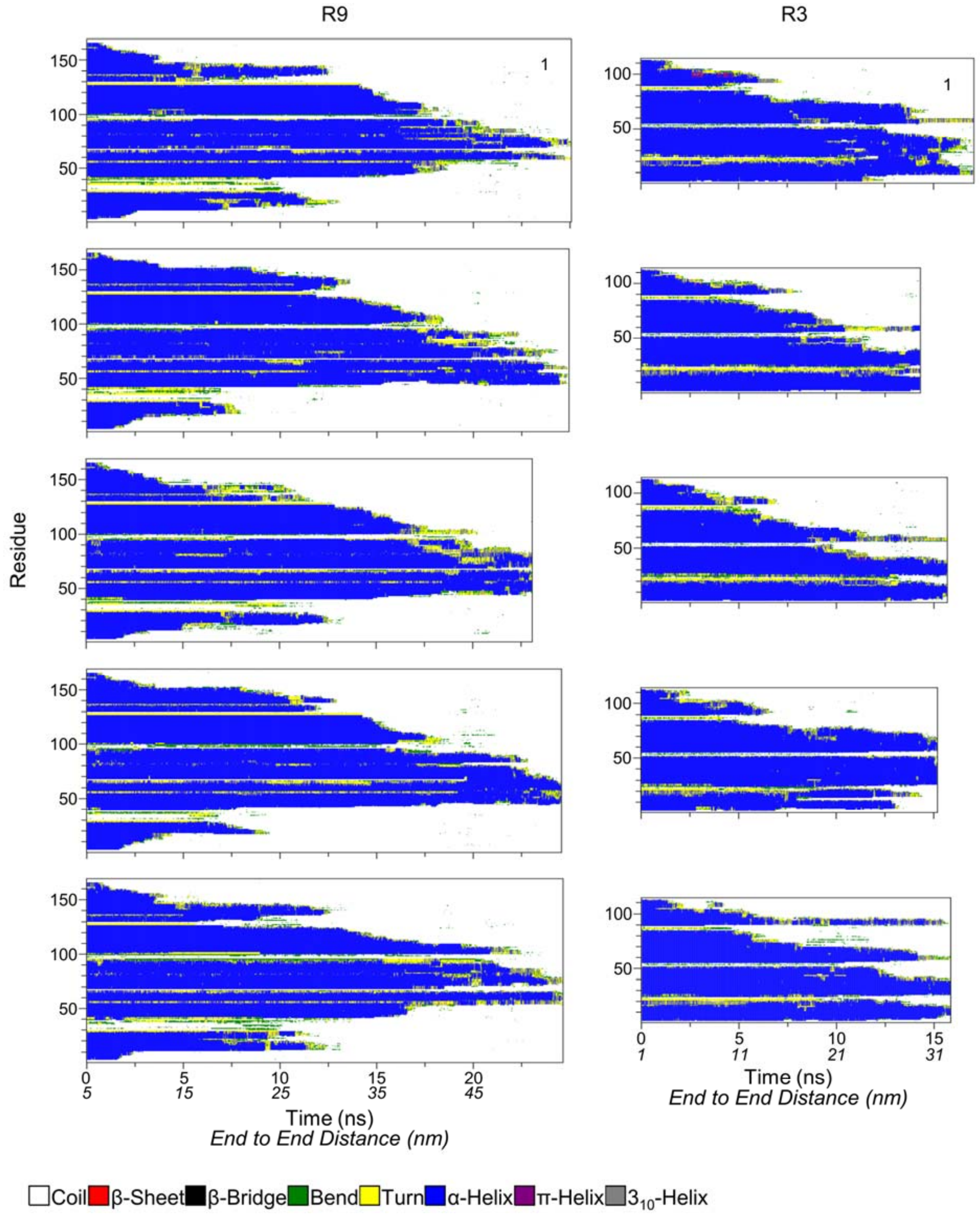

Figure S3. Secondary structure analysis of talin rod (left) R9 and (right) R3 during unfolding in all-atom SMD simulations. The analysis was performed using DSSP, for all five replicas.

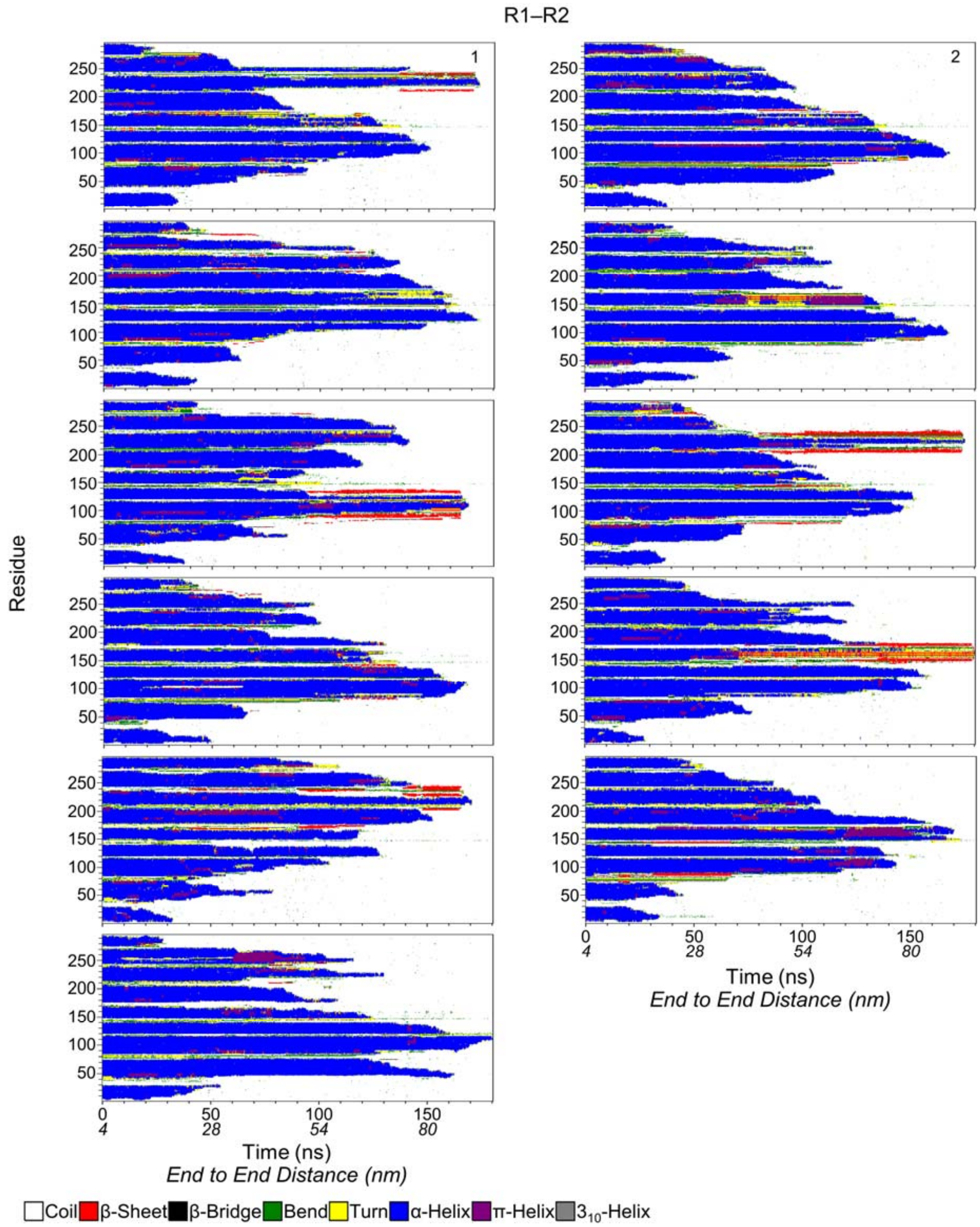

Figure S4. Secondary structure analysis of talin rod R1–R2 fragment during unfolding in BXD simulations. The analysis was performed using DSSP, for all eleven replicas.

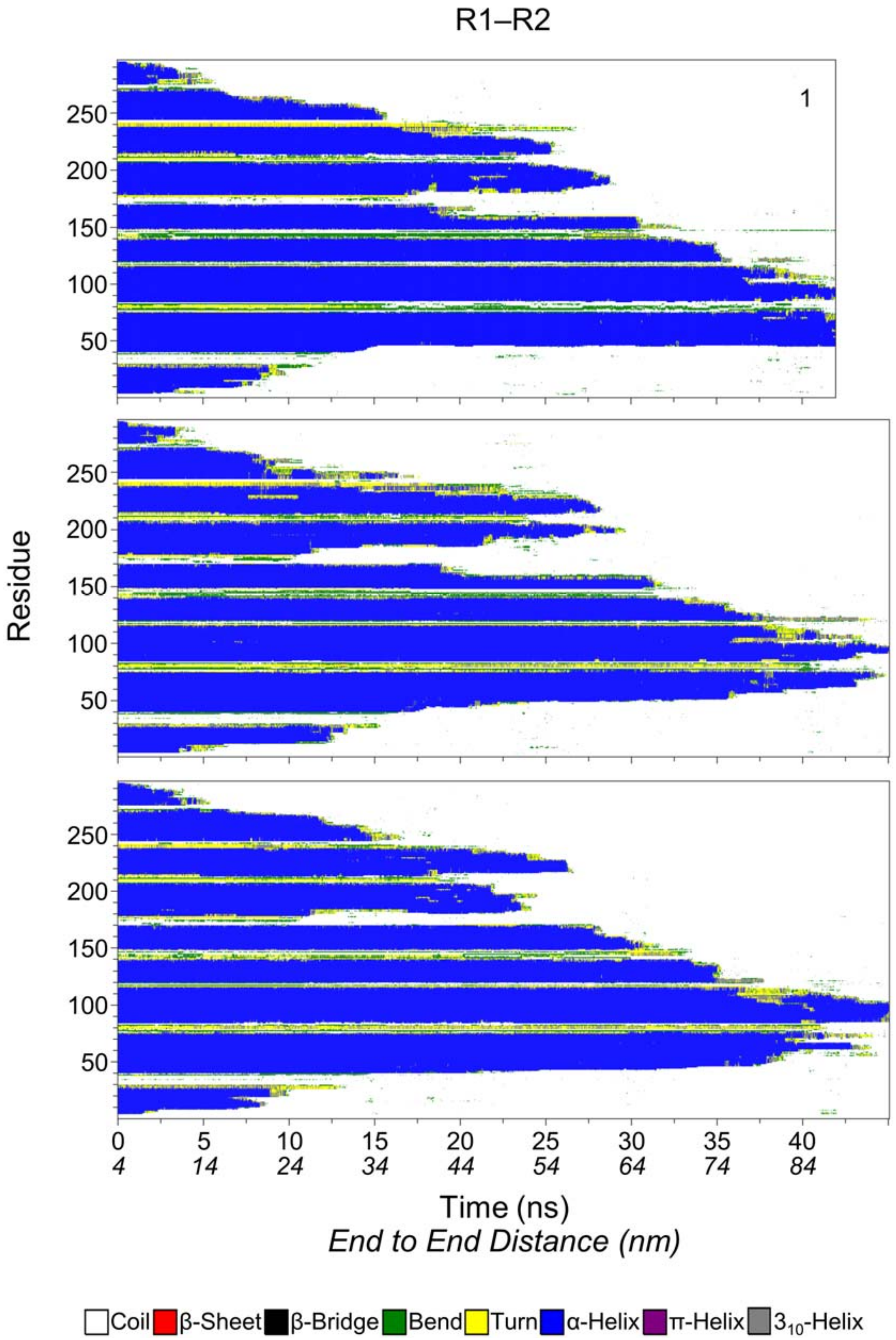

Figure S5. Secondary structure analysis of talin rod R1–R2 fragment during unfolding in all-atom SMD simulations. The analysis was performed using DSSP, for all three replicas.

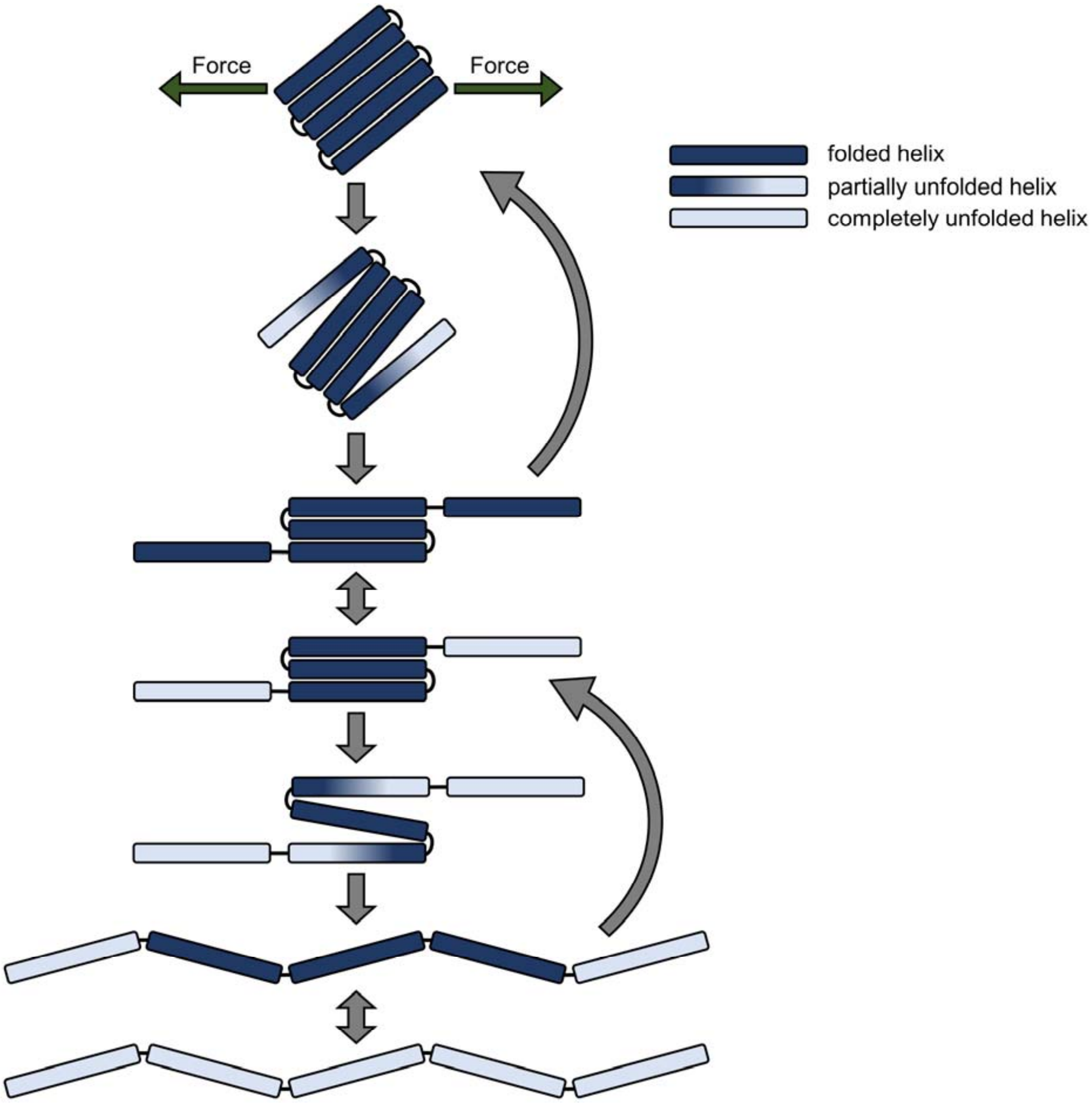

Figure S6. A model unfolding of the individual 5-helix talin rod subdomain. In the absence of force, the subdomain is completely folded. Under force, the terminal helices (namely, H1 and H5) undergo partial unfolding during breaking of the 5-helix structure, but after dissociation from the remaining 3-helix state the secondary structure refolds. As the force increase, the terminal helices undergo complete unfolding, followed by the break of the 3-helix state in the same scenario. In case of force decreasing, the structure refolds.
